# Flexibilizing the Retirement Transition: Why, How and for Whom? Conceptual Clarifications, Institutional Arrangements and Potential Consequences

**DOI:** 10.3389/fsoc.2021.734985

**Published:** 2021-10-11

**Authors:** Simone Scherger

**Affiliations:** Socium Research Center on Inequality and Social Policy, University of Bremen, Bremen, Germany

**Keywords:** flexible retirement, pensions, partial pensions, flexibility, flexibilization, pension systems, pension reform, inequality

## Abstract

In many countries, flexibilizing the retirement transition is seen as an innovative policy which may help to solve some of the problems ageing societies face. The paper aims at specifying what is or can be meant by flexibilizing the retirement transition. The proposed conceptual framework contributes to a better understanding of the potential individual and structural consequences of flexibilized retirement transitions. It spells out four dimensions based on which measures of flexibilization can be differentiated, compared and examined more closely: aggregate vs. individual flexibilization (the latter resulting in gradual retirement), the temporal form and reference of flexibilizing measures, accessibility and eligibility, and financial risks and costs resulting from flexible transitions to retirement. These dimensions of comparison are exemplified by referring to existing measures of retirement flexibilization, in particular wage subsidies and partial pensions. Based on the conceptual argument, some of the potential consequences of flexibilized retirement transitions are discussed critically and in particular with regard to questions of social inequality. As these reflections show, the framework may also help to unpack the policy logic behind flexibilizing retirement transitions, and the very different interests it may serve.

## Introduction

As populations in most Western societies are ageing and public budgets are under strain in many Western countries, pension systems and arrangements regarding the transition to retirement are facing great pressures.^1^ Politicians are thus looking for innovative policy solutions to reconcile competing, sometimes even contradictory aims in old age policies, such as prolonging working lives, reducing public expenditure (or at least preventing its increase), preserving or re-establishing social justice, and avoiding old age poverty. Measures to flexibilize the retirement transition constitute one such policy solution which is assumed to benefit most involved parties. However, although the flexibilization of the retirement transition is on everyone’s lips and figures importantly in many political party programs and declarations of intent, there seem to be as many meanings of flexible retirement as there are political positions, and often what is meant by the term differs widely.^2^

This paper aims at specifying what is usually or can be meant by flexibilizing the retirement transition. I will spell out different dimensions that help to categorize and analyze the different ways in which a flexible retirement transition can be realized. The resulting conceptual differentiation can serve as a tool for describing and comparing existing measures of retirement flexibilization, also between countries or over time, their consequences and their implications with regard to social inequalities. The resulting framework contributes to a better understanding of the different kinds of policy logic behind flexibilizing retirement transitions and the different interests it may serve. I will argue that the answer to the question of whether the promises associated with the flexibilization of retirement can really be achieved, be they longer working lives, saving money, or individual wellbeing, depends on the design of measures and strategies of flexibilization. In many cases, reasonable doubts exist as to the fulfilment of these promises.

The paper adds to the literature that deals with the intersection of individual life courses and social policies. It gives a conceptual and necessarily partial overview of the literature on flexibilized retirement transitions, their implications and consequences. Given the abundance of related literature, it can only cover parts of the quickly evolving discussion which now spans more than two decades. Correspondingly, the limited number of examples of policy measures I give is from a small number of countries, chosen in a pragmatic way, and by tendency representing a European view on life course regulation and pensions.

As a starting point, institutionalized retirement, the retirement transition and the role of pension age in modern welfare states are discussed. Then I sketch the promises that are connected to the idea of flexibilized retirement and outline what flexibilization means in the context of retirement. The following section presents the core of the argument and spells out four different dimensions of measures which can serve to describe flexible retirement transitions: aggregate versus individual flexibilization, the temporal dimension (amongst others in relation to pension age), accessibility and eligibility criteria, and financial costs and risks. After summarizing contested issues around gradual retirement transitions, the final discussion and conclusions point to the gaps of this argument, and consider the prospects of flexibilized retirement transitions critically and with a specific emphasis on social inequalities.

## The Institutionalized Retirement Transition and the Role of Pension Ages

In modern Western (and other) societies retirement is defined as the phase at the end of one’s life that is free from the need to participate in the labor market because one or several old age pension(s) are paid. Early forms of retirement have existed throughout centuries for selected members of society, such as soldiers or civil servants. However, in most countries, retirement as a separate life stage of a considerable length (of at least a few years) has only become a reality for the majority of the population sometime after the Second World War (Kohli, 1987; Thane, 2006). The conditions for this to happen were increasing longevity and a concentration of death in higher ages, as well as the expansion of (public or other) pension systems providing sufficiently high pension incomes which actually allowed the majority of the population to stop working. Consequently, the actual employment rates of older people after pension age decreased to the level of a small minority, albeit with a considerable degree of variation over time and in different countries. The transition to retirement and the life stage of retirement itself thus became predictable and a matter of biographical expectation. Before, the majority of older people had to keep on working as long as they were able to. As their health and skills declined, very often a process of de-skilling into less demanding and lower paid jobs took place. In the case of severe illness and disability preventing paid work, people had to rely on family support or poor relief (Thane, 2006).

A pension system and thus the emergence of a retirement phase are a constitutive part of all Western welfare states, although the concrete institutional arrangements, especially the role of the state in providing pensions, differ widely across countries. A commonality of most of these arrangements is a general statutory pension age which defines the age from which (public) pensions can be received, usually under certain conditions such as having paid contributions for a certain amount of time, or time of residence. As Kohli (1987; Kohli, 2000; also Atchley, 1982) points out, a general pension age at which people withdraw from labor market participation fulfils several collective as well as individual functions in industrialized economies: on the individual level, the pension age serves the cognitive function of enabling individuals to structure and plan their life courses; on the collective level, it helps to organize the labor exit of older (supposedly less productive) workers and succession in a rational way; it organizes the access to old age benefits and thus protects older people from poverty and the necessity to participate in the labor market, and thus, as economists add, serves redistributive purposes (Barr and Diamond, 2010: 78–93); and it has a moral function as a “legitimate” conclusion of working life and form of intergenerational reciprocity (Kohli, 2000: 16; see also Kohli, 1987). In a broader sense and as a constitutive part of modern welfare states, pension systems even contributed to processes of nation-building (Kohli and Arza, 2011: 252).

The transition to retirement consists of two different steps which, at least in its ideal-typical form, happen at the same time: The exit from the labor market and the beginning of pension payments. Although the terms “pension age” and “retirement age” are often confounded, the former refers, strictly speaking, to the age when pension receipt is possible for the first time, while the latter means the withdrawal from the labor market—or sometimes, as “mandatory” or “default” retirement age, the age that prescribes withdrawal from the labor market (see below). The degree to which the ideal-typical concurrence of these two steps (first pension receipt and labor market withdrawal) is realized not only depends on the pension regime considered, with liberal, more individualized welfare regimes usually being characterized by less standardized transitions and higher employment rates before and after pension age (for example Blossfeld et al., 2011). Timing and form of the retirement transition are also subject to historical change. Not only have statutory pension ages been decreasing in the course of the maturation of pension systems. Further institutional as well as historical changes have, at least for a certain time, led to a weakening nexus between institutionalized pension ages on the one hand, and actual transition behavior, i.e., effective pension or retirement ages, on the other. Faced with high unemployment rates, institutional early retirement pathways (as well as parallel policies on company level) were established in many countries which allowed labor exit and the beginning of pension payments before reaching the state pension age and under certain conditions (Kohli et al., 1991). In many countries, early retirement was most widespread in the 1980s and 1990s (for an overview of decreasing effective retirement ages see Latulippe and Turner, 2000: 181). Since then, institutional possibilities of early retirement have been cut back considerably or ceased in most countries (Ebbinghaus, 2006), with the consequence that effective retirement ages have increased again—which can be seen as a de-flexibilization of the retirement transition (Fröhler et al., 2013). Further pension reforms implemented as reaction to perceived demographic pressures and limited public budgets have added to this development, amongst others by reducing the level of pension payments in public schemes, privatizing pension provision, and, importantly, increasing pension ages (Ebbinghaus, 2011; Anderson, 2015).

## Flexibilizing the Retirement Transition

### The Promises of Flexibilized Retirement in the Context of Policies to Extend Working Lives

In many OECD countries, recent waves of pension reform include flexibilizing the retirement transition as an aim, and concrete measures allowing for (more) flexibility have been implemented. Flexibilizing the transition also features prominently in plans for future pension reform in party programs, government reports, and also in recommendations by inter- and supranational bodies and organizations, including the EU and the OECD. However, while the aims and hopes connected to it are high, it is often not clear what exactly the appealing catchword of retirement flexibilization relates to.^3^

Flexibilizing the retirement transition is seen as a way “to contribute to the general increase of working lives” (European Commission/Social Protection Committee, 2007: 2; also European Commission, 1999; Reday-Mulvey, 2000) and to raise the labor market participation of older workers (see also Belloni et al., 2006; Fornero and Monticone, 2007), amongst others in order to avoid potential labor shortages (Eurofound, 2012a: 9). Some even see the flexibilization of the retirement transition as an alternative to increasing pension ages (Bredt, 2008). Prolonging working lives and increasing the labor market participation of older workers are therefore primarily motivated by the wish to contain the costs of pension systems. These are seen to be under pressure because of demographic ageing, but also further factors such as dampened economic growth, deregulated labor markets and unemployment (Pierson, 2001; see also footnote 1).

Over and above the aim of containing public expenditure, flexibilized retirement transitions are also assumed to have positive effects on the level of labor markets, companies and individual actors. Latulippe and Turner (2000: 182) assume, without giving supporting evidence, that partial retirement can “improve worker morale and reduce absenteeism”, and Reday-Mulvey (2000: 54) postulates it can increase productivity per hour. Flexible transitions are also supposed to give “individuals more choice in their retirement transitions” (European Commission/Social Protection Committee, 2007: 2; Fornero and Monticone, 2007; Belloni et al., 2006). As surveys show, many older workers indeed say that they wish to retire gradually (for several countries: Aegon Center for Longevity and Retirement, 2015; for Germany: DGB, 2014). A Eurobarometer survey from 2012 shows that almost two thirds of Europeans find the possibility to combine partial pensions with a part-time job more attractive than full retirement, and roughly one third would like to continue working beyond pension age. Furthermore, more than two thirds think that a lack of gradual retirement options is a main barrier for older people working (European Commission/TNS opinion and social, 2012: 47–48, 74–79). Accordingly, the common perception by individual actors as well as policy makers is that avoiding abrupt “cliff edge” retirement and instead retiring gradually benefits the wellbeing of older workers and future retirees (OECD, 2006: 98–99). According to this view, it can ease psychological adjustment to retirement, and help to adjust work hours in case of health limitations (also Latulippe and Turner, 2000: 181–182).

Consequently, flexibilizing the retirement transition supposedly has the potential to reconcile competing goals of pension reform, such as saving money and allowing a reduction in work hours for those who need it. Following this view, implementing flexibilizing measures and regulations should lead to a win-win situation for all. While it is not the aim of this paper to check these assumptions in detail (but see the section *Contested Issues Around Gradual Transitions to Retirement* for related literature), the following argument offers a framework for evaluating existing measures of flexibilization.

### Defining Flexibilization

When talking about flexibilized (retirement) transitions, it is necessary to distinguish between the *rules and regulations* which organize these transitions on the one hand, i.e., pension regulation, statutory pension ages etc., and *actual transitions* and their timing on the other, in particular effective retirement ages. The actual process of transitioning to retirement has always been more complex than implied by its institutional regulation, which often assumes retirement as a single step of stopping work and starting to receive a pension, or at least very few steps of transitioning. In particular the actual retirement transitions of women have always been (more) complex, whose employment patterns are more discontinuous throughout prime working age and also in the transition to retirement (see for example Loretto and Vickerstaff, 2013; Ní Léime and Loretto 2017). At the same time, retirement processes follow systematic patterns which are not arbitrary and can be compared between different social strata, historical times, countries, or welfare regimes. An important concept to describe these temporal patterns is (de-)standardization (see, for example, Brückner and Mayer, 2005; Scherger, 2009), which relates to both the incidence and temporal uniformity of transitions. The latter includes the order and timing of transitions and their sub-steps, the reversibility and the (un-)ambiguousness of transitions, their aggregate temporal variation and their duration or gradation.

In the following, by the term “flexibilization”^4^ of retirement transitions I mean *intended forms of limited de-standardization*. This flexibilization can be based on purely individual strategies or on institutional measures. It is mostly the latter that the debate around pension and retirement policies refers to. Tendencies of de-standardization in factual (retirement) transitions can, however, also occur despite or “against” institutional regulation, for example as unintended and potentially undesired effects of unemployment in late careers. In this paper, I focus on the institutional level, i.e., I understand as “flexibilization” those pension- and retirement-related regulations or strategies that intentionally aim at making the transition to retirement less temporally fixed and uniform, more pluralized, more varied, more heterogeneous or simply: more flexible, both on the aggregate and individual levels.^5^ Importantly, “flexibilization” is by definition limited in its degree and does not mean a completely individualized and unregulated transition—which would imply the disappearance of any generalized retirement transition.

A flexible retirement transition in this sense relates to the order, timing and organization of the two steps of the retirement transition—withdrawal from work and beginning of pension payments—which do not need to happen completely or simultaneously anymore if the transition is flexibilized. This may lead to a longer and ambiguous transition period between the main working career and full retirement, which some call “partial retirement” (Latulippe and Turner, 2000). More concretely, measures and strategies to flexibilize the retirement transition then relate to the questions of whether and to what extent one can stop working before pension age or continue working after pension age, and of when, how, and under which conditions they can receive a pension. While *institutional regulations* aiming at the flexibilization of retirement transitions (such as partial pensions) offer a defined frame and defined rules with regard to when, how and for whom flexibilized transitions are possible, *purely individual strategies* to flexibilize one’s retirement transition can be applied regardless of legal regulation. These individual strategies of “DIY flexibilization” will be discussed in the following as well because they form an important benchmark for understanding institutional regulation related to the flexibilization of retirement. It is of course not only the state and individual actors who decide upon and shape flexible retirement transitions. Employers, either individual employers or collectively organized employers, also play a crucial role (see Fröhler et al., 2013 for the example of Germany). It is employers who hire and fire older workers before, at or after pension age, offer part-time jobs or not, allow for gradual reductions in work hours or not. Finally, collective representation of workers often plays an important role, as unions^6^ are involved in collective agreements with employers, for example.

In the following, I will describe different dimensions of measures to flexibilize the retirement transition. These dimensions are key to understanding how the state, employers and potentially unions open, restrict or even prevent individual actors’ choice in transitioning flexibly from working (full-time and without receiving an old-age pension) to complete retirement. The concrete examples of flexibilizing measures I refer to are chosen pragmatically from a limited number of European countries and are necessarily only of exemplary character. Furthermore, there is an emphasis on measures connected to the first pillar of public pensions. Corresponding rules may also exist for occupational and private pensions at least with regard to some measures (such as partial pensions).

## Dimensions of Flexibilizing the Retirement Transition

I propose four analytical dimensions in order to systematically describe and examine measures and strategies that flexibilize the retirement transition and that can be applied to all forms of flexible retirement transitions. First and very generally, flexibilization can be related to the *aggregate* level of the retirement transition or to *individual* retirement transitions. The second dimension relates to the question of *when* (i.e., before or after pension age) and in *which temporal patterns* flexibilization is supposed to take place. Third, policy measures related to flexible retirement transitions are often conditional upon the fulfilment of *eligibility criteria* and not *accessible* to everyone. Fourth, strategies and measures to flexibilize the retirement transition differ with regard to who bears the related (potential) *financial costs* or *risks*.

### Dimension 1: Aggregate Versus Individual Flexibilization

A flexibilized retirement transition can either relate to the timing of the transition as observed on the aggregate level, or to the individual level. Flexibility on an (exclusively) *aggregate* level implies that individual retirement transitions happen in one clear-cut step, but that the age of this transition varies between individuals. By contrast, flexibilization on the *individual* level refers to *stepwise, gradual* or *phased* individual transitions. The latter meaning often takes center stage in current debates and suggestions around the flexibilization of the retirement transition.

The most important example of flexibilization on the *aggregate* level is early retirement. Measures allowing early retirement have become less important in debates around pensions in recent years, but are still relevant, especially against the background of (further) increasing statutory pension ages. Collective actors who criticize recent pension policies and the prolongation of working lives still see early retirement as an essential form of flexibilizing the retirement transition, especially in the case of incapacity to work or disability. Unions in many countries demand financially protected early retirement options for those who are ill or disabled (for Germany and the United Kingdom: Hagemann and Scherger, 2016). In its terminological thesaurus, the International Labour Office defines “flexible retirement” as the “option given to retirees to choose the age at which they retire (usually within certain limits)” (ILO 2020). Institutionalized early retirement, i.e. rules allowing people to retire and receive a pension before reaching regular pension age, were very common in many Western pension regimes (for overviews see Kohli et al., 1991; Ebbinghaus, 2006), especially in conservative welfare states. The explicit aim of many of these policies was to alleviate labor market pressures (and to improve unemployment statistics) in times of high unemployment—seemingly offering a win-win situation for all involved parties. In the heyday of these regulations, claiming pensions based on these pathways was often not connected to deductions in pension payments. In recent decades, such early retirement pathways without pension deductions have been phased out.^7^ While early retirement is still possible in many countries, early retirees have to accept deductions in pension payments. A special case of early retirement are (full or partial) incapacity pensions granted to those who are not able to work anymore, or only in part-time, due to health reasons. In most countries (and often in contrast to the past), they are conditional upon strict health tests and accessible to younger people as well. For older people, who constitute the majority of their claimants, they can also function as a flexible transition to old age.

Allowing the deferral of pension receipt beyond pension age is the second way of flexibilizing the retirement transition on the aggregate level. Pension deferral is often rewarded by higher pension payments and seen as a measure to incentivize longer working lives (Eurofound, 2012b). In the United Kindom, for example, deferral of the state pension is rewarded by a permanent increase in the amount of the pension of around 5.8 per cent for every year after pension age that the pension is not claimed (Department for Work and Pensions, 2021); in Germany, this permanent increase is 0.5 per cent per month (6 per cent per year) (Czepek and Weber, 2015; for further countries see Eurofound, 2012b: 51–52).

A far-reaching and also symbolically highly relevant form of flexibilizing pension age on the aggregate level is the abandonment of a “normal” or “regular” pension age. An example for this can be found in the Swedish pension system. Since the reform in the 1990s, the public Swedish earnings-related pension scheme only defines a lowest possible pension age of 61, and each year that someone retires later than this is rewarded by an estimated 10 per cent increase in annual pension income (OECD, 2015: 352–355; Halleröd, 2015: 110). For the universal guarantee pension for low income earners, however, a pension age of 65 applies (which is expected to be increased in the years to come), and this guarantee pension is tested against the amount of the earnings-related pension that someone would have received at the age of 65—thus institutionally still “expecting” a normal pension age of 65 in this tier of the pension system.

Strictly speaking, deductions for early retirement and rewards for late retirement (i.e., after statutory pension age) in pension systems which still have a statutory pension age are equivalent to such a “corridor” of pension ages. However, the symbolic significance of a clear and fixed pension age may be high as it fulfils the (cognitive) function of biographical orientation (see Kohli 1987; Kohli, 2000).

Flexibilization on an *individual* level implies a gradual, stepwise or phased transition to retirement.^8^ This means, in general terms, that the withdrawal from the labor market, pension receipt and age are combined in unusual ways, with the consequence that the retirement transition is only completed in several steps and in a longer period of time. Usually such a gradual transition involves reducing work hours at some point. While continued full-time work and at the same time receiving a pension can be seen as a gradual transition as well, I will, in the following, concentrate on measures and strategies which in one way or the other imply reduced work hours, i.e., part-time work. This may also refer to a new job. Employment which forms a ‘“bridge” between main (full-time) career and full retirement, and which may span beyond pension age, is often related to as “bridge employment”, especially in the American literature (for example, Alcover et al., 2014). The two most important explicit policies which support reducing work hours and thus working part-time in preparation for full retirement are wage subsidies for older people who are close to pension age, and the payment of partial (or even full) pensions. In the following, I will focus on these measures as prime examples of policy measures aiming at gradual retirement transitions on the individual level.

### Examples of Measures Aimed at Gradual Transitions to Retirement

*Partial* (i.e., reduced) *pensions* are an important way to retire gradually. The International Labour Organization (ILO), e.g., defines “partial retirement” as the “combining of part time employment with receipt of a reduced pension” (ILO 2020). Rules for drawing partial pensions exist in many countries, with the Scandinavian countries having a longer tradition of offering partial pensions (see, for example, Ginsburg, 1985; for further examples from other countries see; Belloni et al., 2006; Eurofound, 2016). The payment of (statutory) partial pensions is independent from where the older worker is employed. Often the wish to reduce work hours may imply having to find a new, part-time job.

In Sweden, for example, at the minimum age for pension receipt in the earnings-related public pension, 61, one can also claim a partial pension (of 25, 50, or 75 per cent) which is possible in both parts of the pension, the pay-as-you-go NDC-scheme (income pension) as well as the funded part (premium pension), or in a combination of both (Palmer, 2004; see also Lindquist and Wadensjö, 2011). No upper age limit or earnings limit apply, and further pension rights can be accrued if contributions are paid based on continued work. The full pension is recalculated upon full retirement. The German earnings-related social insurance pension for instance now allows for flexible partial pension receipt—of a flexible percentage between 10 and 99 per cent of the full pension—for everyone who can already claim a pension, either under one of the few remaining institutional early retirement paths (then with deductions on the partial pension) or under the regular statutory old age pension. Before reaching regular pension age (but not after), earnings limits are applied which are in most cases derived from the partial retiree’s full wage in the last 15 years.^9^ In the time before reaching regular pension age, further pension claims are accrued based on the pension contributions for the part-time job; after pension age, the same applies, and there is an additional accrual corresponding to the (in this case partial) reward for pension deferral (Deutsche Rentenversicherung, 2020a; Deutsche Rentenversicherung, 2020b). So far, partial pensions are only rarely received in Germany (Deutsche Rentenversicherung, 2020c: 197), although they have been flexibilized in 2017.

*Wage top-ups* of different kinds, especially if subsidized by the state, can also enable older workers to work part-time in order to transition to retirement gradually. In this case, older workers usually stay with their employer, reduce their work hours to part-time work in the prospect of retirement, and at least parts of their wage loss are compensated. The German “Altersteilzeit” (literally: old age part-time) can be cited as an example of such a legal measure, which had mainly labor-market related aims when it was introduced in 1996 (based on another version of such a regulation existing earlier). The related law created a frame for regulations on the company level or on the level of collective agreements; the possibility of such transitions continues to exist, but the wage top-ups are not subsidized anymore. Under certain conditions, the framework allows older workers from the age of 55 to reduce their work time flexibly until retirement, for example to half-time, and still get roughly 70 per cent of their old wage, plus an even higher share of pension contributions (Fröhler et al., 2013: 57–64). The wage top-up was, until the end of 2009, subsidized by the state if an unemployed person or someone who had just finished their education was newly employed for the job of the person making use of the possibility of “Altersteilzeit”. Most people and companies who benefited from the law, however, did not use it for a stepwise reduction of work hours, but in its “block” version, meaning that the employee continued to work full-time with reduced wages in a first phase, and then completely left work in a second phase, in which, like in the part-time model, parts of their wage were still paid. Thus the regulation was actually mostly used as a path to early retirement. A similar scheme with state-subsidized wage top-ups exists in Belgium (Albanese et al., 2015: 8–11), although eligibility criteria are being tightened, especially by raising the age of eligibility. France is another example of a country where a similar scheme existed which was ceased in 2005 (Hallé and Jolivet, 2007; see also Latulippe and Turner, 2000: 192).

Besides partial pension receipt and (subsidized) wage top-ups for older part-time workers, further flexibilizing measures mainly organized on the level of collective agreements or of single companies include the use of long-term working time accounts (Wotschack and Hildebrandt, 2007). Such long-term accounts can enable individual workers approaching retirement, but also in other phases of life, to reduce their work hours while continuing to receive their full wages, if they have “saved” the appropriate amount of time (and money) on their account. While the use of such accounts is at the discretion of employers, they nonetheless need to be regulated on the national level (for the German example see Fröhler et al., 2013: 70–78). Finally, occupational pensions may also offer possibilities of gradual retirement if they can be paid out early.

Apart from measures offered by the state and/or the employer, reducing work hours is of course also possible outside of such schemes. In these cases, the individual workers can for example fall back on their savings, the financial support of others, or even state benefits other than pensions (such as social assistance) to compensate the wage loss, although the latter are usually conditional upon further criteria or may be subject to means-testing. All these possibilities, however, still presuppose that the employer allows the reduction of work hours or that a new job with part-time hours is available. Continuing work in a part-time job is of course also possible after pension age. This can be combined with full or partial pension receipt, if pension receipt is not completely deferred.

### Dimension 2: Timing and Temporal Patterns of Flexibilization

The second dimension by which flexible retirement transitions can be characterized relates to the question of *when* and in *which temporal patterns* flexibilization is supposed to take place. First and foremost, this refers to the temporal association with pension age: Flexibilizing measures or strategies can relate to the time before state pension age, and allow for an earlier starting point of the transition to retirement, or to the time after state pension age, or to both. Typically, different temporal patterns also imply different conditions and consequences. Regarding early retirement, there is usually a lower age boundary for earliest pension receipt (with the exception of incapacity pensions), while an upper age for pension deferral only exists in a minority of countries (for EU-countries: Eurofound, 2012b: 51–52). For subsidized wage top-ups such as those described above stricter age-related rules normally apply. In most countries there has been a shift with regard to the timing flexibilizing measures aim for. While attention to measures geared towards flexibilized transitions spanning *past* pension age has grown and more of such measures have been introduced, measures beginning a longer time before pension age have been at least restricted in access and/or financially penalized, if not abolished, especially if they include complete early retirement (Ebbinghaus, 2006).^10^

Other temporal characteristics of flexibilizing measures concern the question of whether they are limited in their duration and have a latest end, such as full retirement at pension age for the wage top-ups described above. Rules related to timing (i.e., age) and duration affect how long the “hybrid” zone between the main working career and full retirement lasts, and how many steps it consists of. Theoretically, several measures and strategies can be combined, leading to an even longer transition to gradual retirement. For example, an older worker could continue working part-time and receive a partial pension from shortly before pension age until one year after, then reduce his/her work hours further while receiving a full pension, to finally retire completely at age 70. However, realizing such pathways to retirement, especially if they involve several steps of reducing one’s work hours (i.e., phased retirement in a strict sense), may be difficult because of the accessibility of part-time jobs and the eligibility for partial pensions (see below).

An issue closely related to the timing and the temporal patterns of gradual transitions is the existence of *default retirement ages*, which force older people to give up their job at a certain age. Framing European legislation, more concretely the European Directive on employment equality (2000/78/EC), in principle bars general default retirement ages.^11^ This, for example, contributed to the abolition of the British default retirement age in 2011 (Department for Business Innovation and Skills, 2011) and increased the chances of continued employment for older people. Still, forms of mandatory retirement (ages) continue to exist in many countries, if not on the national level, but on the levels of collective or company agreements, or for specific occupations. Germany is a typical example of a country in which retirement ages are still ubiquitous on these levels, and may hinder continued employment beyond state pension age (Mahlmann, 2011: 82–86; O'Dempsey and Beale, 2011: 68, 75). On a more general level, pension systems differ with regard to the question in how far regulations but also the related cultural set-up allow for a clear differentiation between pension age and retirement age—a disentanglement which is a precondition for organizing flexible retirement transitions. The idea that a pension age cannot and should not imply a (simultaneous) mandatory retirement age has traditionally been more common in Anglo-Saxon liberal welfare states, with the United States being one of the earliest examples of abolishing mandatory retirement, in 1986. With regard to cultural norms regarding old age, ideal pension ages and extended working lives, the existence of different “work-retirement cultures” has been empirically corroborated, which impact actual old age transitions (Jansen, 2018).

### Dimension 3: Eligibility for and Accessibility of Flexible Retirement Transitions

The third analytical dimension of flexibilized retirement transitions concerns the question of how accessible measures of flexibilization actually are. The related measures are often not accessible to everyone and can be conditional upon the fulfilment of eligibility criteria. The more regulated and financially supported measures and strategies of flexibilization are, the more eligibility criteria tend to apply. Receiving a partial pension is usually not only conditional upon a minimum age, but also a minimum period of contributions, in Germany for example the same contribution periods as for full pension receipt. If a partial or full pension is claimed due to incapacity (or disability), minimum contributions may be lower, but health or disability tests will apply. Whether an older worker can access schemes like subsidized wage top-ups depends on the one hand on general conditions, such as contribution periods. On the other hand, it depends on the approval of the employer, the corresponding regulations on the company level or on the level of collective agreements. In coordinated market economies, collective agreements can play a prime role in organizing and regulating flexible retirement transitions, and in protecting employees from potentially negative consequences of such transitions. However, such schemes are often only accessible to selected (longer-term) core employees.

Beyond these institutional eligibility criteria especially for those who transition to retirement flexibly from their old job, the other essential condition for being able to retire gradually through working part-time is the availability of a suitable job. If future retirees want to reduce their work hours from full-time to part-time and stay in the same job, their employer has to allow for such a reduction. Although there are no corresponding systematic empirical studies, sketchy evidence indicates that such a reduction is not necessarily welcome in many sectors and branches. Many people wishing to retire gradually therefore choose to or have to work in a different job in order to realize their plans. However, finding a job around pension age is difficult in many countries, as stereotypes and age discrimination are rife (Krekula and Vickerstaff 2017: 20–30), especially with regard to re-employment as opposed to retention (Schmitz, 2015). Additionally, labor legislation may impede the employment (or retention) of older people especially after pension age.^12^ For all of these reasons some older people accept downward mobility, in particular after pension age, as a part-time job is often not available in their former qualified occupation, and more part-time jobs are often available in low-paid service jobs requiring no or low qualifications.

Empirical evidence on those who work beyond pension age in part-time and thus realize a form of gradual retirement shows that doing so seems particularly easy for well-qualified people including many self-employed (see for example Scherger, 2015a; also Alcover et al., 2014). At the same time, in some countries, low service, part-time jobs seem to be easier to find for older people (Lain, 2012) who may use them for their individual strategies of gradual retirement. As they do not have an employer, self-employed have more choice in reducing their work hours in order to retire gradually—which is underlined by the high share of self-employed among all workers around and after pension age in most countries (Eurofound, 2012b: 38–41; contributions in Scherger, 2015a). At the same time, however, this high share may indicate that some formerly employed workers choose to become self-employed, amongst others because they are faced with difficulties to find a new job, or that some long-term self-employed have low pensions and less access to (partial) pensions so that they have economic reasons to delay their retirement.

Both, eligibility rules for measures of flexibilization and availability of suitable jobs, cannot be overestimated in their importance for whether flexibilized retirement transitions are possible, and for whom this is the case. Put more generally, many labor market- and employer-related “push”-factors into complete retirement (Ebbinghaus, 2006: 11–19) are also negatively connected to the availability of gradual retirement transitions. However, another, more variable factor is as crucial as eligibility criteria and the availability of (part-time) jobs: the financial incentives for and risks of flexible retirement transitions.

### Dimension 4: Financial Costs and Risks of Flexibilized Retirement Transitions

A “key strategy” of pension reform in Europe has been “to operate via incentives: incentives to work, incentives to save, and incentives to retire later” (Kohli and Arza, 2011: 4). This also applies to flexible retirement, with later transitions often being rewarded financially, and earlier (partial) retirement and pension receipt being connected to financial penalties in the form of pension reductions. Thus a fourth and crucial dimension in which strategies and measures to flexibilize the retirement transition vary is the question of who bears the *financial costs* or the *financial risks* of a flexibilized and gradual transition to retirement (or vice versa, who reaps the financial rewards). This question is essential for understanding who is able to realize flexible retirement transitions and what consequences this may have for their later full retirement in terms of financial resources. Saving financial costs by prolonging working careers is one central goal of promoting flexibilized transitions to retirement, and to be realized, these savings must be generated in some way. Generally speaking, such costs can be borne by the individual, by the collective (of pension contributors in the case of contributory pensions or tax payers for tax-financed pensions)—or by the employer in the case of occupational pensions.

There are different ways of looking at the financial dimension of flexible retirement transitions. From a *(macro-)economic point of view* and focusing on pension systems and state budgets, actuarial neutrality or fairness of flexible retirement transitions are an aim and a benchmark based on which corresponding measures are assessed (for an economic definition see Bridges and Disney, 2005: 49; Simonovits, 2003). The economic line of thinking also implies that possibilities of flexible (earlier) transitions will always be seized by individual actors if there are financial incentives to do so, which often implies costs on the collective level, as in the case of partial pensions paid before pension age. The aim of actuarial neutrality means that any measure should be conceived in a way so that, for example, partial pension receipt before regular pension age does ultimately not lead to higher costs in the form of higher lifetime pension payments. Leaving aside the question whether this should be the most important criterion of evaluation, this is only possible if early (partial) pension receipt involves relative reductions in pensions, or if combining the partial pension with working indeed leads to a longer career with longer pension contributions, compared to what would have happened without the partial pension. With regard to existing measures of flexibilization, attempts of corresponding economic analyses doubt that they have really led to actuarially neutral pensions or to overall reduced costs (see next section). In a similar way, it is a debated question whether and in which proportion cost savings through working beyond the pension age (should) benefit the individual actor, for example in the form of a higher pension. Finally, actuarial neutrality often builds on the wrong assumption that life expectancy is distributed evenly; to be a more accurate approximation, the underlying modelling would have to include the fact that many early (partial) retirees have a much lower life expectancy than those who work longer or even beyond state pension age (see for example Simonovits, 2003).

From an *individual point of view*, by contrast, the more temporally proximate and concrete financial consequences of a gradual retirement transition will matter more than the relatively abstract actuarial fairness of institutionalized (partial) pension receipt. As the economic and sociological literature show, many individual actors have a low level of financial literacy and know little about finances in general and their own financial (retirement) arrangements (Hershey et al., 2012: 410–419; Barr and Diamond, 2010: 38–44). This also means that they often only tend to plan for the short term or at maximum for the medium term both financially and in other regards (Rowlingson, 2002). While actuarial pension calculations are based on predictions of life expectancy for certain groups, these are only based on averages, and the length of their individual life (and that of their partners) is still unpredictable for individual actors. Faced with this uncertainty, long-term calculations and planning are difficult to realize. Thus most older workers will usually not calculate whether retiring gradually will lead to financial gains or losses in their lifetime. In many cases, individual actors will have no or very limited choice regarding their options of retiring and/or continuing work. In the case of a relatively high scope of action, people will tend to think counterfactually with regard to the short and perhaps the medium term; being able to keep a certain level of income (and thus living standard) will be more relevant than accumulated lifetime income. Being faced with long-term uncertainties also implies that more proximate factors may shape the decision on whether someone continues working or not: Health, labor-market or household-related circumstances, in particular limiting ones, will be important here, and unexpected events may make any plan obsolete (Burtless, 2006; Loretto and Vickerstaff, 2013).

While early pension receipt is nowadays connected to deductions in pension receipt, in order to make it actuarially neutral and less attractive, deferring pension receipt is rewarded because people pay contributions for a longer time and (or) later receive their pension for a shorter time. If done before reaching statutory pension age, retiring gradually and thus reducing working time is connected to considerable financial risks which can be attributed to different parties. The measures of wage subsidies or partial pensions described above are the prime institutionalized ways in which wage losses through reducing work hours can fully or partly be compensated. To which degree these costs are compensated and thus borne by the state (or employers) depends on the level of the wage subsidies or the partial pension, and also on the degree to which future pension payments are reduced because of lower pension contributions. When partial pensions are drawn early (i.e., before pension age), deductions will usually apply to the part of the pension that is drawn early, and they will very often be applied permanently. Such potential permanent deductions may be compensated in the long run if working is continued, possibly beyond pension age, and leads to the accrual of further pension claims. Drawing an occupational pension before pension age may also cushion a reduction in income, as might generous redundancy payments, but again the pension will be reduced. As this opportunity is distributed very unevenly across potential retirees, only those with good workplace pension schemes or/and higher wages can afford this form of gradual retirement before pension age without sizable losses in pension income.

Working hours can of course also be reduced without receiving a (partial) pension or a wage substitute—in this case, flexibilization is organized in a “DIY” way and without obstacles due to eligibility criteria, apart from the possibility to reduce work hours. However, the costs of this are solely borne by the individual, in the form of a lower current income, but also of lower later pension claims, depending on the pension system. In flat-rate pension systems such as the British one, reducing work hours may have no or only little effect on later pension claims in the first pillar, as long as the income is above a certain threshold, whereas in earnings-related systems such as the German one, the reduction in pension payments may be considerable. The same applies to most occupational or private pensions. This will be crucial for individual actors in their decisions as it impacts the retirement lifestyle they can afford.

With regard to the possibility to draw a partial pension *at or after* pension age, the risk of immediate income loss for the individual will be low. Vice versa, it can of course be asked who benefits from the additional pension contributions that are made through working. The individual financial consequences of flexible retirement transitions in the sense of prolonged careers depend on the possibility to accrue further pension claims through working (see also Latulippe and Turner, 2000: 186–187). If no pension is drawn, further pension accrual should be common, also beyond state pension ages. However, when working hours are reduced, pension contributions might be low in earnings-related pension schemes, or the employment may even be so marginal that no pension contributions are paid at all—leading to lower future pension payments. With regard to partial pensions, different rules apply in different countries.

Finally, rules for earning extra besides pension payments can be looked at in a similar way. They indirectly affect whether working is financially worthwhile. If, for example, strict earnings limitations^13^ apply while receiving an early pension and the pension is tested against earnings, this might disincentivize work and thus gradual retirement, as the potential income from working is reduced (Latulippe and Turner, 2000: 184–186). In many European countries, rules for earning extra apply to the time before regular state pension age, i.e., when drawing a pension early, but not anymore after (Eurofound, 2012b: 51–52). The abolition of all earnings limits, which is often discussed as a measure to increase individual flexibility, is for example opposed by some German political actors, as this would, in their eyes, undermine the wage-substituting function of pensions.^14^

## Contested Issues Around Gradual Transitions to Retirement

Despite the popularity of the general concept of flexibilization, it is not at all clear whether flexibilizing the retirement transition is generally beneficial—and if yes, for whom. The consequences of flexibilized retirement transitions are debated and their benefits questioned on several levels. These consequences need to be studied in more detail, differentiating different conditions and forms of flexibilization, kinds of outcomes and social groups affected.

*First*, on the *individual level*, access to a gradual retirement transition is unequally distributed. The concrete design of flexibility in the retirement transition, in terms of accessibility and eligibility and of financial costs and risks, determines who is able to realize flexible retirement transitions and then actually benefit from their advantages. If the related financial costs fall on the individual, partly or entirely, this limits their degree of choice with regard to realizing options of flexible retirement. These costs are thus another criterion of access to gradual or phased retirement transitions. Additionally, there may also be situations in which in particular women have to reduce their work hours because of private obligations to provide long-term care—and in which gradual retirement itself is based on constraints.

Financially, gradual retirement starting with part-time work before pension age can go together with serious consequences for individual old age income, in the form of permanent pension deductions, wage decreases, undesired dissaving for old age etc. This applies especially to “DIY”-flexibilization and flexibilization due to other constraints (for example care needs) which are not framed by supporting measures like wage subsidies or partial pensions. As poor incomes and insufficient provision for old age are closely related and often affect those in more strenuous jobs and in ill health, using possibilities to retire gradually may be connected to additional financial disadvantages for them, while continuing work full-time may affect their health adversely. On the other hand, the individual financial effects of working part-time may be beneficial if working part-time allows staying in employment for longer than working full-time would have done, at least under certain conditions—but so far there is little evidence of such a positive financial balance.

Of course, financial consequences should not be the only measure of evaluation, and perhaps not even the main one. However, *second*, it is also still an open question whether people transitioning to retirement flexibly benefit in terms of individual wellbeing. Reitzes and Mutran (2004) or de Vaus et al. (2007) do not find clear benefits in wellbeing (health, positive and negative affect, wellbeing, post-retirement attitudes) for people who transitioned gradually to retirement compared to those who underwent an abrupt transition. Similarly to the wellbeing effects of retirement in general (van Solinge, 2013) or the effects of employment past retirement age (Lux and Scherger, 2017), it is less the general temporal pattern of the transition than the further circumstances of retirement which determine its outcomes in terms of wellbeing: health, the control over the transition the individual has (de Vaus et al., 2007) or further circumstances of working and other life domains. Especially the third and fourth dimension of the design of flexibilized retirement transitions described above will thus moderate their impact on individual wellbeing; positive (or at least no negative) outcomes are most likely in cases in which a gradual transition, its exact shape and timing are the consequence of individual choice and do not go together with considerable income losses. By contrast, if gradual retirement means being forced to give up one’s main career, taking on a part-time job that is not paid well or only offers mediocre or bad working conditions, and accepting downward mobility, the probability of unfavourable wellbeing outcomes is higher. Measures allowing partial retirement may thus imply “legitimating precarious conditions of employment for older workers” (Latulippe and Turner, 2000).

A *third* and connected critical issue on the individual level is the complexity and transparency of the transition of retirement. As discussed above, a fixed pension age (also) serves the cognitive function of enabling individuals to structure and plan their life courses. Offering measures to flexibilize retirement transitions increases the complexity of retirement transitions. In particular, the financial consequences of a flexibilized retirement transition can be highly opaque. Biographical planning with regard to the retirement transition is much less systematic and deliberate than the design of policies presupposes, and often follows, as in the case of unexpected events, an ad hoc logic. Biographical orientation may be further complicated by flexibilized pension transitions, and this applies even more to “DIY”-gradual transitions and those building on private pensions. The more steps a gradual retirement transition involves (with phased retirement in a strict sense meaning the “progressive limitation of hours of work for older workers”, ILO, 2020), the more complicated it is to organize the transition, financially and otherwise. Thus, “phased” retirement transitions in a strict sense will only be realizable for few people, and probably not those who would benefit most. In a wider sense these potential negative side effects of flexibilizing the retirement transition chime with the general debate on flexibility and flexible lives (for examples from a life course perspective see Guillemard, 2005).

*On a structural and systemic level*, the benefits of flexibilized retirement transitions are not unambiguous either. *First*, the discussion so far shows that gradual retirement transitions bear the danger of increasing inequalities and cumulative (dis-) advantages in old age. Favourable arrangements are often only open to those who are in an advantaged position anyways (for the German example see the analysis of Kerschbaumer, 2009), who probably benefit from existing measures more often (Latulippe and Turner, 2000: 183) or are able to organize flexible transitions for themselves more easily without supporting policies. Vice versa, potentially harmful forms of flexibilized transitions probably affect those more often who are in less favourable positions and have a lower life expectancy from the start. Just as prolonged employment careers in general bear the risk of rising inequalities (see for example Scherger, 2015b: 18–20), gradual transitions may add to this. Eligibility criteria connected to health have the potential to benefit those who are disadvantaged^15^, and counteract such rising inequalities, if the resulting pensions are adequate. Thus flexible retirement arrangements cannot and should not replace early retirement opportunities for those in bad health (for a corresponding example of inequality-related consequences of tightened eligibility criteria for disability pensions in Sweden see Kadefors et al., 2019). At the same time, successful measures which enable gradual retirement for people with health limitations hinge on the availability of suitable part-time and less strenuous jobs, underlining the importance of employers’ willingness to offer such jobs and related human resources practices.

Even from a purely economic viewpoint, *second*, the benefits of flexibilized retirement transitions in the form of a positive balance of financial costs and benefits is often not confirmed. Albanese et al. (2015) and Hermansen (2015), for example, show for the Belgian and the Norwegian contexts, respectively, that reduced work hours before full retirement do not have a significant (preventing) effect on the probability to take early full retirement, and thus do not save pension expenses (for an overview of similar studies with mixed and context-dependent results, see Eurofound, 2016: 27–34). For gradual retirement regulations, such as partial pensions, to contribute to saving money, they need to be offset by longer (part-time) careers of those who use such schemes—at least longer than in the counterfactual case without partial retirement. Or they need to be offset by others who prolong their career for example by working beyond pension age. The exact economic effects of working part-time before retirement depend on a whole range of factors, including the hours worked, and depend highly on (national) context (Eurofound, 2016: 31–34). Apart from the normative question of whether economic considerations should be the main measure for evaluating retirement arrangements, a desirable scenario without rising inequalities would probably imply a higher degree of redistribution from those privileged workers who can prolong their career considerably, be it by full-time or by part-time employment, to those whose early (partial) retirement can then be cross-financed by the resulting savings. Such a system, however, implies complex mechanisms of redistribution in a collectively organized pension system.

*Third*, labor market effects of gradual retirement especially when reaching beyond usual pension age can be debated in a similar way as effects of prolonging working lives in general. Some critics see the call for flexibilized transitions as part of a neo-liberal employer strategy to increase (cheap) labor supply and keep wages low, in particular in countries where labor shortages because of demographic ageing are looming (see Macnicol, 2015, especially chapter 2; Krekula and Vickerstaff 2017).

*Fourth* and finally, the wider consequences of a declining relevance of age boundaries and the ensuing decreasing significance of (workfree) retirement are subject of controversy. Retirement and the “right” to a workfree retirement are closely related to the legitimation of welfare states, although the exact shape of this connection depends on the concrete welfare tradition. While a carefully regulated window of flexibility may not imply questioning (mostly) workfree retirement as such—which has always been a normative ideal—critics of flexibilization see some of its forms as a first step of putting an end to the social achievement of retirement. Such a critique is more pronounced in countries whose pension system used to ensure the maintenance of living standards, such as the German one, and in the case of actors who favor a stronger welfare state, such as unions (see Hagemann and Scherger, 2016). These actors take particular issue with regulations which undermine the function of pensions to maintain achieved living standards, for example the loosening of rules for earning extra or rules for extra pension accrual; they fear that such measures lead to the expectation that everybody must work longer. In this perspective, such smaller rules are more than mere technical details. They take on a symbolic role, pointing to the intended meaning of pensions—as main income source securing one’s living standard or as one source of income amongst several ones. Giving up these markers of pension age increases pressures to work longer and threatens the protective function of retirement and pension ages.

## Discussion and Conclusions

This paper aimed at giving an overview of what it can mean to flexibilize the retirement transition, of different dimensions of the corresponding flexibilizing measures and strategies, and of their potential consequences. Focusing on conceptual issues, the examples given for flexibilization are necessarily fragmentary and incomplete. In order to evaluate measures to flexibilize the retirement transition, the institutional context in its entirety needs to be considered, in particular the whole package of regulations related to old age. This does not only encompass pensions and the concrete pension mix (including occupational and private pensions and other savings for old age), but also other old age related benefits, especially means-tested social assistance, health care and provisions for long-term care—as they all affect financial costs or risks of retirement transitions. For example, health care insurance through their employer plays an important role in work decisions of older Americans below the age of eligibility for Medicare. Additionally, home ownership impacts the financial needs of people in old age.

The design of measures flexibilizing the retirement transition and thus their consequences will cluster along the lines of welfare regimes or varieties of capitalism; this assumption could inspire empirical research on flexibilizing measures and on their individual and structural consequences. Studying these consequences is, however, methodologically challenging, as, for example, selection into flexibilized transitions needs to be disentangled carefully from their effects in comparison to what would have been the case if these transitions had not been flexibilized.

A further complicating factor is the fact that the above deliberations (as many institutional regulations) had an idealized full-time career as their starting point, which does not apply to (most) women (and some men) whose careers are characterized by interruptions and shorter or longer spells of part-time employment. More or less discontinuous careers affect and probably relativize the meaning of full-time retirement and thus of a flexibilized retirement transition, and they also complicate the described aspects of flexibilization in their regulation and organization, in their subjective perception and in their analysis. To explore more deeply this relationship between earlier careers, flexible transition patterns and the surrounding regulations is a further important subject of future research.

The discussed measures of flexibilization and their expansion can be understood as part of more general developments in the realm of old age, pensions and welfare regimes: The shift of responsibility to individuals and the privatization and marketization of life course risks (see for example Meyer et al., 2007; Ebbinghaus, 2011) which is critically discussed in the broader frame of the “neo-liberalisation” (Macnicol, 2015) or “activation” of old age in “flexible capitalism” (van Dyk, 2014; Krekula and Vickerstaff 2017). In many of the politically pushed ways to design them, flexibilizing measures individualize and privatize the organization of the transition to retirement and also its risky consequences, implying a shift in responsibility towards the individual actor. This regards the financial, but also all other consequences of flexibilized transitions. At the same time, shifting ascriptions of responsibility ignore heterogeneity among workers and obscure the fact that individual choice with regard to the retirement transition is actually often (very) restricted, in particular because of individual health, (insufficient) economic resources and limited labor market opportunities. Replacing relatively fixed ages of first pension receipt with flexible rules regulating if, when and how much pension payments an older person receives, seems on the one hand to open up more possibilities and a higher degree of choice in the transition. On the other hand, these rules are usually tied to financial incentives and to eligibility criteria, so that flexible and gradual transitions are unequally accessible, which also pertains to the availability of appropriate, well-paid (part-time) jobs. Thus the seemingly higher degree of choice is in contrast to the actually very often limited scope of individual action. Just like flexibility with regard to working times or to careers, potential benefits and disadvantages depend very much on the design and conditions of flexibilized arrangements, on who determines them, and the surrounding context and institutions. Vulnerable individual actors will usually only have little power in these processes, unless they are well protected through according regulation. While flexibility is an enticing concept for all political actors, different actors tend to favor very different designs of measures to flexibilize the transition, with very diverse potential consequences.

If enforced perverse redistribution from those with low to those with high life expectancy (see Haan et al., 2020) and rising inequalities due to prolonged careers and flexible retirement transitions are to be prevented, a uni-directional shift of responsibility to individual actors should be avoided. More engagement from the state and from employers is needed to flexibilize the retirement transition in a way that is more likely to benefit not only a privileged group—although the latter outcome may be acceptable under circumstances when it is at least cost-neutral or even collectively beneficial. Collective bargaining and collective agreements may play a crucial role in negotiating good regulation. Employers need to facilitate a gradual retirement transition by allowing people to reduce their work hours when approaching retirement or by offering (more) part-time workplaces for older people. Measures like long-term working time accounts or job-sharing may further enhance actual possibilities of gradual retirement. The state can facilitate gradual transitions by offering transparent partial pensions and further rules that do not penalize working longer or in combination with pension receipt. To whom these possibilities should be open and which financial consequences they should have for later full pensions, are crucial questions with regard to the individual and collective outcomes of such rules. The answers to these questions need to balance flexibility and transparency as well as individual choice and collective responsibility in a way that takes into account the heterogeneity of workers and fits the surrounding regulations. Economic outcomes cannot be the only, and perhaps not even the main criterion by which to evaluate measures of flexibility—and strategies based on cost-saving only will probably fail.

While completely fixed pension ages may not always be adequate considering the heterogeneity of older people, their careers, their needs and family-related or other obligations, they nonetheless offer a stable frame of biographical orientation and social protection for those who cannot work any longer. Enabling everyone to work up to pension age, in good-quality work and in an occupation they trained for, is an important step in prolonging working lives. Flexibilizing the retirement transition can probably only be a complementary measure in this process. Evidence so far indicates that it cannot solve the underlying health- and labor-market related problems, and that reasonably generous early retirement arrangements for those in bad health continue to be highly relevant and legitimate. A completely flexibilized retirement transition may even threaten the protective and cognitive functions of pension ages and end up costing more in terms of means-tested benefits or of rising inequalities and old age poverty.

## Footnotes

1 While population ageing is one reason for rising public budgets at least to some degree in many countries, there are other reasons as well and the exact role of population ageing is contested, as is the question of whether the problem of population ageing is not greatly exaggerated by some political actors in order to justify cutting social policy spending.

2 The idea for this paper emerged from interviews with experts on old age, work, and pensions who also talked about measures to flexibilize retirement transitions. These interviews exemplify the mentioned variety of interpretations of the term (see Hagemann and Scherger, 2016).

3 Relatively vague concepts with positive connotations, such as flexibilization, are particularly useful in politics, as they are open for projections and different interpretations but still seem to relate to important shared values. Apart from “flexibility” (which is used in different policy contexts) other examples for this are “fairness” or “intergenerational justice”.

4 The term flexibilization has its roots in debates on labour market flexibilization; the meaning of these two kinds of flexibilization is at least similar. Flexible retirement transitions are also substantially connected to flexibilized labour markets in several ways, for example in that older people can be seen as a flexible labour market reserve.

5 For examples of the study of actual retirement transitions and their flexibilization, see Blossfeld et al. (2011) and Buchholz et al. (2011).

6 The importance of unions varies widely between countries, and even in countries with well unionized workers, union coverage is far from complete.

7 Women’s lower state pension ages, which have been or are phased out in most countries where they existed, can be seen as a special case of these early retirement regulations.

8 Further terms that are used here are partial retirement, or, more rarely, “progressive” retirement (Eurofound, 2001).

9 These earnings caps were suspended in the two years of the COVID-19-pandemic, 2020 and 2021.

10 This is also reflected in the cited older overviews of policies to flexibilize the retirement transition (see for example Latulippe and Turner, 2000; Reday-Mulvey, 2000).

11 Fixed retirement ages may, however, still be legal in cases where they are well justified, as in the case of specific occupations or because of certain conditions on the labor market.

12 For example, German employers for a long time were reluctant to retain their employees beyond pension age because legally, they were obliged to give them permanent contracts if they had a permanent contract before. Corresponding legal exceptions were introduced in 2014.

13 Strict earnings limits usually apply in the case of means-tested (old age) benefits, which disincentivizes working longer for those claiming these benefits. Vice versa, if continuing work is possible, this may also discourage from claiming the benefits. Applying for the benefits may be complicated and connected to the experience of stigma, and earning income through paid work can be experienced as staying financially independent and self-sufficient (for the United Kingdom see for example Radford et al., 2012).

14 This was the position of the Confederation of German Trade Unions (DGB) and of an organization representing the rights of pensioners, disabled people, patients or those in need of care in the interviews reported in Hagemann and Scherger (2016).

15 See also Haan et al. (2020) who show that the German public pension insurance (over time increasingly) benefits those with higher incomes if their higher life expectancy is considered in the underlying calculations.
