# Erratum: Flexibilizing the Retirement Transition: Why, How and for Whom? Conceptual Clarifications, Institutional Arrangements and Potential Consequences

**DOI:** 10.3389/fsoc.2021.845393

**Published:** 2022-02-24

**Authors:** Frontiers Production Office

**Affiliations:** Frontiers Media SA, Lausanne, Switzerland

**Keywords:** flexible retirement, pensions, partial pensions, flexibility, flexibilization, pension systems, pension reform, inequality

Due to a production error there were multiple corrections to be made to the reference list. Further minor corrections have been made to the text.

The publisher apologizes for this mistake. The original version of this article has been updated.

